# Convergent functional networks of intrinsic activity alterations in temporal lobe epilepsy and their molecular correlates

**DOI:** 10.3389/fnmol.2026.1909269

**Published:** 2026-08-10

**Authors:** Liang-Liang Ma, Ling-Ling Yang, Bao-Zhen Zhou, Qian Gao, Bo-Chao Zheng, Hu-Cheng Yang, Gen-Di Wang, Ting-Ting Yang

**Affiliations:** 1 Department of Pediatrics, Binhai County People’s Hospital, Yancheng, China; 2 Department of Pediatrics, Binhai Clinical College, Yangzhou University Medical College, Yancheng, China; 3 Department of Neurology, Yancheng Third People’s Hospital, Affiliated Hospital 6 of Nantong University, Yancheng, China; 4 Department of Radiology, Yancheng Third People’s Hospital, Affiliated Hospital 6 of Nantong University, Yancheng, China; 5 Department of Radiology, Binhai Maternal and Child Health Hospital, Yancheng, China

**Keywords:** brain network, functional connectivity network mapping, gene expression, neurotransmitter, temporal lobe epilepsy

## Abstract

**Background:**

Temporal lobe epilepsy (TLE) is increasingly recognized as a network disorder, yet reported regional intrinsic neural activity alterations from resting-state fMRI studies remain spatially heterogeneous. This study aimed to determine whether these heterogeneous alterations converge onto a shared functional network and to characterize its normative transcriptomic and neurochemical correlates.

**Methods:**

Using a coordinate-based network mapping approach (functional connectivity network mapping, FCNM), we delineated a common brain network functionally connected to regional intrinsic neural activity alterations reported across 20 published neuroimaging studies. The robustness of the resulting network was assessed in an independent cross-scanner validation connectome and across different seed sizes. We further characterized this TLE-related network by correlating its spatial topography with microscale gene expression data from the Allen Human Brain Atlas (AHBA) and with normative neurotransmitter receptor and transporter distributions derived from the JuSpace toolbox.

**Results:**

Twenty studies comprising 345 foci of regional intrinsic neural activity alteration in TLE were included. The FCNM analysis revealed that heterogeneous regional alterations in TLE converged onto a common functional brain network. This network exhibited the greatest spatial overlap with the default mode network (DMN), while also showing substantial overlap with the limbic network (LN). Transcriptomic analysis revealed that the network’s topography was spatially correlated with gene expression profiles significantly enriched in adaptive immune response pathways, particularly antigen processing and presentation. Neurochemically, the TLE-related network exhibited a significant positive spatial correlation with the distribution of the 5-hydroxytryptamine receptor 1A (5-HT1A).

**Conclusion:**

Our findings reconcile previously inconsistent reports of regional intrinsic neural activity alterations in TLE by demonstrating their convergence onto a shared brain network, primarily the DMN and LN. By linking this TLE-related network to specific normative transcriptomic and neurochemical signatures, we propose a multi-scale neurobiological framework for the disorder. These findings should be interpreted as spatial associations based on normative datasets rather than direct evidence of disease-specific molecular alterations. This framework reframes TLE from a collection of disparate regional changes toward a core network dysfunction with distinct molecular correlates, thereby opening new avenues for targeted, network-based interventions.

## Introduction

Epilepsy is a prevalent neurological disorder affecting over 70 million people worldwide (Thijs et al., 2019). Temporal lobe epilepsy (TLE) is the most common type of drug-resistant focal epilepsy (Sathe et al., 2022), and frequently causes varying degrees of cognitive impairment, including memory deficits, naming difficulties, executive dysfunction, and attentional disorders (Bell et al., 2011; Giovagnoli and Avanzini, 2000; Titiz et al., 2014). Furthermore, TLE has a profound impact on the quality of life and represents a significant socioeconomic burden (Busch et al., 2023; Forthoffer et al., 2025). Although seizures often originate from temporal lobe structures, accumulating evidence indicates that TLE involves distributed functional and structural networks rather than a single isolated epileptogenic region (González Otárula and Schuele, 2020; Kramer and Cash, 2012). Therefore, understanding how regional abnormalities are organized at the network level is essential for clarifying the neurobiological mechanisms of TLE and for developing network-informed biomarkers and therapeutic strategies.

Resting-state functional magnetic resonance imaging (MRI) measures, including the amplitude of low-frequency fluctuation (ALFF), fractional ALFF, regional homogeneity (ReHo), and their dynamic variants, have been widely used to characterize regional intrinsic neural activity in TLE (Song et al., 2022). However, findings on intrinsic neural activity alterations remain inconsistent across studies. For example, some studies have shown increased activity in the cingulate cortex (Chen et al., 2012), precuneus (Song et al., 2022), angular gyrus (Mao et al., 2023), and hippocampus (Song et al., 2022) in TLE, whereas others have reported decreased activity in the cingulate cortex (Chen et al., 2012), precuneus (Song et al., 2024; Wang et al., 2020), and angular gyrus (Zhu et al., 2018). Conversely, one study reported no significant local functional alteration in TLE (Chang et al., 2022). These discrepancies may reflect differences in clinical subtype, seizure lateralization, disease duration, medication exposure, sample size, MRI acquisition, and analytic pipelines. More importantly, they suggest that regional intrinsic activity alterations in TLE may not be adequately understood as isolated local abnormalities.

A network localization framework provides a useful approach to address this challenge. Recent evidence suggests that neuropsychiatric symptoms and disease-related abnormalities may be localized more effectively to distributed brain networks than to isolated anatomical regions (Boes et al., 2015; Darby et al., 2019; Fox, 2018). This network-centric view posits that seemingly disparate regional abnormalities may in fact be part of the same shared functional network. Network mapping approaches have successfully identified common brain networks underlying various neuropsychiatric conditions despite heterogeneous regional findings (Cheng et al., 2025; Schaper et al., 2023; Yao et al., 2025). Applying this network perspective to TLE may help resolve previous inconsistencies regarding local activity changes and offer a more biologically plausible model of its neural substrates. Functional connectivity network mapping (FCNM) offers a powerful and increasingly validated methodology for this purpose (Cheng et al., 2025; Li et al., 2025; Zhang X. et al., 2024). FCNM employs large-scale normative connectome data, often sourced from projects like the Human Connectome Project (HCP; Glasser et al., 2016), to integrate discrete, region-specific findings into a unified functional connectivity network framework.

Beyond identifying the implicated brain networks, clarifying their normative molecular correlates is essential for a comprehensive understanding of TLE. The neurobiological basis of local alterations in TLE is increasingly thought to be associated with patterns of gene expression and neurotransmitter systems (Aronica and Gorter, 2007; de Lanerolle et al., 1992). Recent genetic studies have revealed that TLE is characterized by substantial genetic heterogeneity (Salzmann and Malafosse, 2012). Advanced transcription-neuroimaging approaches offer insights into the molecular and genetic foundations of altered brain networks (Zhang D. et al., 2024). By spatially correlating macroscale brain network maps with microscale spatial transcriptomics data, such as the Allen Human Brain Atlas (AHBA), researchers can uncover gene expression signatures associated with specific network abnormalities and explore their genetic basis (Ji et al., 2021; Negi and Guda, 2017; Xu et al., 2023). Furthermore, integrating these network abnormalities with neurotransmitter system atlases derived from PET or SPECT imaging can further elucidate the underlying chemoarchitectural basis. This multi-level integrative analysis bridging macroscale networks and microscale molecular mechanisms has proven highly effective in studying conditions such as subjective cognitive decline (Lan et al., 2025). However, whether regional intrinsic neural activity alterations in TLE converge onto a shared network with specific normative transcriptomic and neurochemical correlates remains unclear.

To address this gap, we integrated coordinate-based FCNM with normative transcriptomic and neurochemical mapping. First, we tested whether published coordinates of regional intrinsic neural activity alterations in TLE converge onto a common functional network using large-scale normative resting-state connectomes. Second, we examined whether the spatial topography of this TLE-related network was associated with brain-wide gene-expression patterns derived from the AHBA. Third, we assessed its spatial correspondence with normative neurotransmitter receptor and transporter distributions using JuSpace. We hypothesized that heterogeneous regional intrinsic activity alterations in TLE would converge within networks implicated in temporal-limbic and cognitive dysfunction, particularly the default mode network (DMN) and limbic network (LN), and would show spatial associations with immune-related transcriptomic profiles and serotonergic neurochemical architecture.

## Materials and methods

### Literature search and selection

Following the PRISMA guidelines, we conducted a comprehensive search of PubMed, Embase, and Web of Science to identify studies reporting alterations in regional intrinsic neural activity, including ALFF, fALFF, dynamic fALFF, ReHo, and dynamic ReHo, in patients with TLE, published between January 1, 2000 and March 5, 2026. Search keywords and inclusion/exclusion criteria are detailed in the Supplementary material. In addition, the bibliographies of relevant review articles and meta-analyses were manually screened to capture all potentially eligible studies beyond the primary search. To avoid data duplication arising from overlapping patient samples across publications, only the study reporting the largest sample size and the most comprehensive information was retained for analysis. All coordinates from prior studies were converted to MNI space using established tools^1^ if originally reported in Talairach space. When additional information was needed, the corresponding author of each included study was contacted via email. Two investigators (M. L. L and Y.L.L) independently performed the literature search, selection and data extraction. Discrepancies were resolved through discussion with a third investigator (Y.T.T) until consensus was reached. A flow diagram of the study selection process is presented in Supplementary Figure 1.

### Discovery and validation datasets

This study included two independent cohorts: the Human Connectome Project (HCP) S1200 dataset as the discovery cohort and the Southwest University Adult Lifespan Dataset (SALD) as an external cross-scanner validation connectome. The discovery cohort comprised 1,093 healthy adults (594 women; mean age, 28.78 ± 3.69 years; range, 22–37 years), whereas the validation cohort included 329 healthy adults (207 women; mean age, 37.81 ± 13.79 years). Demographic details for both cohorts are provided in Supplementary Table 1. HCP data were acquired using a 3T Siemens Connectome Skyra MRI scanner, providing high-resolution images suitable for detailed analyses. The full details regarding the sample have been reported in previous studies (Qiu et al., 2025; Van Essen et al., 2013). The Institutional Review Board of Washington University in St. Louis, MO, USA, approved the HCP protocol, and each participant provided written informed consent. For the SALD dataset, resting-state fMRI data were obtained on a 3T Siemens Trio. Exclusion criteria comprised MRI contraindications, current psychiatric or neurological disorders, psychiatric medication use within the past 3 months, pregnancy, or any history of head injury. Rs-fMRI acquisition parameters for both the discovery and validation datasets are summarized in Supplementary Table 2.

### Rs-fMRI data acquisition and preprocessing

Resting-state fMRI data were preprocessed with the SPM12 software and the DPABI toolbox. The HCP data are publicly available at https://www.humanconnectome.org/study/hcp-young-adult/data-releases, SPM12 software at https://www.fil.ion.ucl.ac.uk/spm/, and the DPABI toolbox at https://rfmri.org/DPABI. To ensure signal stability, the first 10 volumes of each scan were discarded. Slice-timing correction and head-motion correction via realignment were then performed. All participants exhibited minimal head motion (maximum translation <2 mm; maximum rotation <2°). The data was processed by regressing out nuisance covariates such as linear drift, Friston-24 motion parameters, signals from white matter and cerebrospinal fluid, and the global signal. Additionally, timepoints with excessive motion (framewise displacement > 0.5 mm) were included as regressors. Following regression, the data were band-pass filtered (0.01–0.1 Hz). The functional images were then normalized to Montreal Neurological Institute (MNI) space using transformation parameters derived from each participant’s T1-weighted image and spatially smoothed with a 6-mm full-width at half-maximum (FWHM) Gaussian kernel.

The HCP resting-state fMRI data underwent minimal preprocessing, including echo-planar imaging gradient distortion correction, motion correction, field bias correction, spatial transformation, normalization to MNI space, and artifact removal via independent component analysis (ICA) + FIX (Glasser et al., 2013). In addition to the minimal preprocessing in HCP, several additional procedures were carried out using the DPABI software. To further explain, nuisance variables like the global, white matter, and cerebrospinal fluid signals were removed through regression. Then, the data were band-pass filtered (0.01–0.1 Hz). Finally, the data were spatially smoothed with a 6 mm FWHM Gaussian kernel.

### FCNM analysis

We applied the FCNM approach to determine whether the reported regional intrinsic neural activity alterations in TLE mapped onto common brain networks. The analysis comprised the following steps: First, 4-mm radius spheres were created at each coordinate of a contrast and then merged together to form a combined seed mask specific to that contrast, referred to as the contrast seed, following previous FCNM research (Cheng et al., 2025; Song et al., 2025; Zhang X. et al., 2024). Next, using rs-fMRI data from the 1093 healthy HCP participants, we computed a whole-brain functional connectivity map for each individual by correlating the mean time series of the seed mask with all other brain voxels (Pearson’s r, Fisher-Z transformed). A group-level FC map was then generated via a one-sample *t*-test on the individual maps, identifying regions with consistently positive connectivity with the seed. We focused exclusively on positive FC, as the interpretation of negative FC remains debated (Murphy and Fox, 2017; Murphy et al., 2009). These group-level t-maps were thresholded (voxel-wise FDR, *P* < 0.05) and binarized. Finally, all binarized connectivity maps were overlaid to create a network probability map, which was then thresholded at 60% overlap – a previously applied FCNM threshold (Mo et al., 2024; Peng et al., 2022)–to delineate the final TLE-related functional alteration network. The sensitivity of the results to this threshold was further examined in robustness analyses. Because the aim of this study was to identify common networks connected to reported abnormal regions, coordinates reflecting both increased and decreased intrinsic activity were combined in the primary analysis.

### Association with canonical brain networks

To enhance interpretability, we analyzed the spatial relationships between the identified TLE-related network and eight established canonical brain networks. The seven cortical networks comprised the visual network, SMN, dorsal attention network (DAN), ventral attention network (VAN), LN, FPN, and DMN, as defined by Yeo et al. (2011). The Human Brainnetome Atlas (Fan et al., 2016) was utilized to delineate the subcortical network. The subcortical network primarily comprised the amygdala, hippocampus, basal ganglia (including the caudate nucleus, putamen, globus pallidus, nucleus accumbens), and thalamus (Fan et al., 2016). We quantified the spatial relationships by determining the percentage of overlapping voxels between the TLE-related network and each canonical network, compared to the total number of voxels in the respective canonical network. Moreover, we used the newly developed Network Correspondence Toolbox (NCT; Kong et al., 2025) to assess the correspondence between our TLE alteration networks and standard canonical networks. By applying Dice coefficients and spin permutation testing, the NCT provides a robust framework for determining the statistical significance of spatial similarity between user-defined regions and existing atlas labels. Because the FCNM-derived TLE-related network extended into the cerebellum, we further characterized cerebellar involvement using a cerebellum-specific atlas. Specifically, cerebellar voxels within the TLE-related network were mapped onto the Buckner 7-network cerebellar parcellation (Buckner et al., 2011).

### Validation analyses

To test the robustness of our results, we replicated the FCNM analyses using the independent validation dataset (the cross-scanner SALD). We then repeated the FCNM analysis again using spheres with radii of 1-mm and 7-mm to assess the impact of seed size. To further assess whether our findings depended on the chosen overlap threshold, we repeated the convergent functional network analysis at two additional thresholds (50% and 70%), again using the HCP dataset and a 4-mm seed radius. We also ran separate FCNM analyses for coordinates showing increased and decreased intrinsic activity as a secondary analysis, and used the NCT to characterize their involvement in canonical networks.

### Transcriptomic analysis

Gene expression data and preprocessing. Regional gene expression data were obtained from the Allen Human Brain Atlas (AHBA^2^), comprising microarray data from six postmortem donors (Supplementary Table 3). A recently developed analytical pipeline was used to process gene expression profiles of over 20,000 genes across 3,702 spatially distinct brain tissue samples (Arnatkeviciute et al., 2019). Detailed processing steps are provided in the Supplementary materials.

### Neurotransmitter analysis

To clarify the relationship between the TLE-related network and chemoarchitectonic organization, we analyzed spatial correlations with 29 PET-derived neurotransmitter transporter and receptor maps from JuSpace version 1.5^3^. These maps included neurotransmitter receptor, transporter, and synthesis-capacity distributions across multiple neurotransmitter systems, including serotonergic, dopaminergic, glutamatergic, GABAergic, cholinergic, noradrenergic, opioid, and cannabinoid systems. To match the spatial resolution and sampling of the FCNM map to that of the neurotransmitter maps, both the FCNM t-map and all PET/SPECT maps were resliced into the same MNI space and parcellated using the neuromorphometrics atlas provided with JuSpace, from which mean regional values were extracted after excluding all white-matter and cerebrospinal-fluid regions. Spearman rank correlations were then computed between the regional FCNM values and each neurotransmitter map. The statistical significance of the correlation was assessed through a permutation test of 5,000 iterations in all analyses, adjusted by false discovery rate (FDR) with a significance level of *p* < 0.05.

## Results

### Included studies and sample characteristics

After a systematic literature search and screening (Supplementary Figure 1), 20 studies comprising 345 reported foci were included. These studies enrolled 1,299 participants, including 688 patients with TLE and 611 healthy controls. The mean age of participants generally ranged from the late 20s to the early 30s, whereas one study focused on a pediatric or adolescent cohort. Reported mean disease duration varied substantially, ranging from 0.24 years to more than 14 years. Cognitive assessments were reported in a subset of studies, most commonly using the Mini-Mental State Examination (MMSE) and the Montreal Cognitive Assessment (MoCA). The most commonly used imaging measures were ALFF, fractional ALFF (fALFF), and ReHo, which were often reported in combination. Eighteen of the 20 studies were performed on a 3.0 Tesla (T) MRI scanner, with the other two studies using a 1.5T scanner. The MNI space was the predominant coordinate system, and only two studies used Talairach space for normalization. The sample and imaging characteristics of the studies included are summarized in detail in Table 1.

**TABLE 1:** Sample and imaging characteristics of the studies included in the study.

Study	Sample size (TLE/HC)	Mean age (TLE/HC)	Disease duration (year)	Cognition (TLE)	Modality/ analysis	Scanner Field Strength (T)	Coordinate space	Foci
Huang et al., 2024	32/33	29 (23, 35)/ 25 (23, 37)	7.00 (3.00, 13.00)	NA	fALFF, ReHo	3.0T	MNI	2
Song et al., 2022	57/42	28.77 ± 11.26/ 26.79 ± 9.02	6.91 ± 8.04	MMSE 28.25 ± 1.86 MOCA-B 24.47 ± 3.56	ALFF, fALFF, ReHo	3.0T	MNI	30
Wei et al., 2016	30/30	30.40 ± 10.60/ 28.60 ± 7.60	8.24 ± 7.06	VIQ 60.94 ± 14.87 PIQ 49.76 ± 10.16 FIQ 96.71 ± 16.40	ALFF	3.0T	MNI	9
Zhang et al., 2010	R:26, L:24/25	R:25.7 ± 7.1, L: 23.3 ± 7.7/25.3 ± 5.8	8.8 ± 5.5	NA	ALFF	1.5T	MNI	48
Zhang et al., 2015	R:26, L:21/32	R:29.5 ± 10.3, L: 27.8 ± 8.1/30.3 ± 9.1	R:10.5 ± 9.3, L:12.6 ± 8.0	NA	ALFF	3.0T	MNI	30
Ji et al., 2013	23/23	27.0 ± 8.5/25.7 ± 8.7	10.8 ± 8.1	NA	ALFF	3.0T	MNI	15
Mankinen et al., 2011	21/21	11.7/NA	2.5	NA	ReHo	1.5T	Talairach	4
Mao et al., 2023	35/33	29.54 ± 8.76/29.52 ± 8.68	8	NA	ReHo	3.0T	MNI	27
Singh et al., 2020	44/40	32.29 ± 10.9/33.17 ± 10.8	NA	MMSE 29.5 ± 0.87	ALFF	3.0T	MNI	20
Song et al., 2024	51/53	28.00 ± 11.98/28.74 ± 9.72	5.48 ± 7.55	MMSE 28.50 ± 1.57 MOCA-B 25.05 ± 3.29	ALFF, ReHo	3.0T	MNI	20
Yang et al., 2022	28/30 19/30	32.86 ± 11.11/35.80 ± 13.01 38.11 ± 12.04/35.80 ± 13.01	10 (7.25, 16.5) 15 (8, 21)	MoCA 25.29 ± 2.34 23.32 ± 2.38	ALFF, fALFF, ReHo	3.0T	MNI	38
Wang et al., 2020	26/26	28.86 ± 6.99/29.31 ± 6.42	11.48 ± 5.87	NA	ALFF, ReHo	3.0T	MNI	25
Zhao et al., 2020	R:18, L:14/48	R: 24.4 ± 6.0, L: 23.6 ± 5.4/ 22.4 ± 2.2	R: 12.1 ± 8.1, L:12.1 ± 4.4	NA	ReHo	3.0T	MNI	25
Zeng et al., 2013	10/15	34.4 ± 10.6/35.5 ± 14.6	NA	NA	ReHo	3.0T	MNI	26
Zhang et al., 2020	16/17	29.63 ± 6.98/27.67 ± 4.97	14.19 ± 3.60	MMSE > 24	ALFF	3.0T	MNI	1
Chen et al., 2012	16/17	34.0 ± 7.8/34.9 ± 6.9	0.24 ± 0.125	NA	ALFF	3.0T	Talairach	5
Zhou et al., 2021	35/33	26.49 ± 5.22/25.6 1 ± 4.59	8.64 ± 5.73	NA	fALFF	3.0T	MNI	3
Zhu et al., 2018	17/21	29.0 ± 6.7/24.9 ± 3.9	4.3 ± 1.2	NA	ALFF, ReHo	3.0T	MNI	10
Qin et al., 2025	60/30	31.62 ± 9.82/ 29.47 ± 7.86	10.98 ± 7.51	MoCA 25.60 ± 3.03	dALFF	3.0T	MNI	1
Song et al., 2023	39/42	29.51 ± 12.72/ 26.79 ± 9.02	5.39 ± 7.88	NHS3 8.92 ± 3.4 MMSE 28.46 ± 1.57 MOCA-B 25.05 ± 3.29	dfALFF, fALFF, ReHo, dReHo	3.0T	MNI	6

TLE, temporal lobe epilepsy; HC, healthy control; MOCA, Montreal Cognitive Assessment; NHS3, National Hospital Seizure Severity Scale; MMSE, Mini-Mental State Examination; NA, not available; ALFF, amplitude of low-frequency fluctuation; fALFF, the fractional ALFF; dfALFF, dynamic fractional ALFF; ReHo, regional homogeneity; dReHo, dynamic regional homogeneity; MNI, Montreal Neurological Institute; L, left; R, right; T, Tesla; FIQ, Full-Scale Intelligence Quotient; PIQ, Performance Intelligence Quotient; VIQ, verbal intelligence quotient.

### Convergent functional connectivity associated with regional intrinsic neural activity alterations in TLE

**FIGURE 1:**
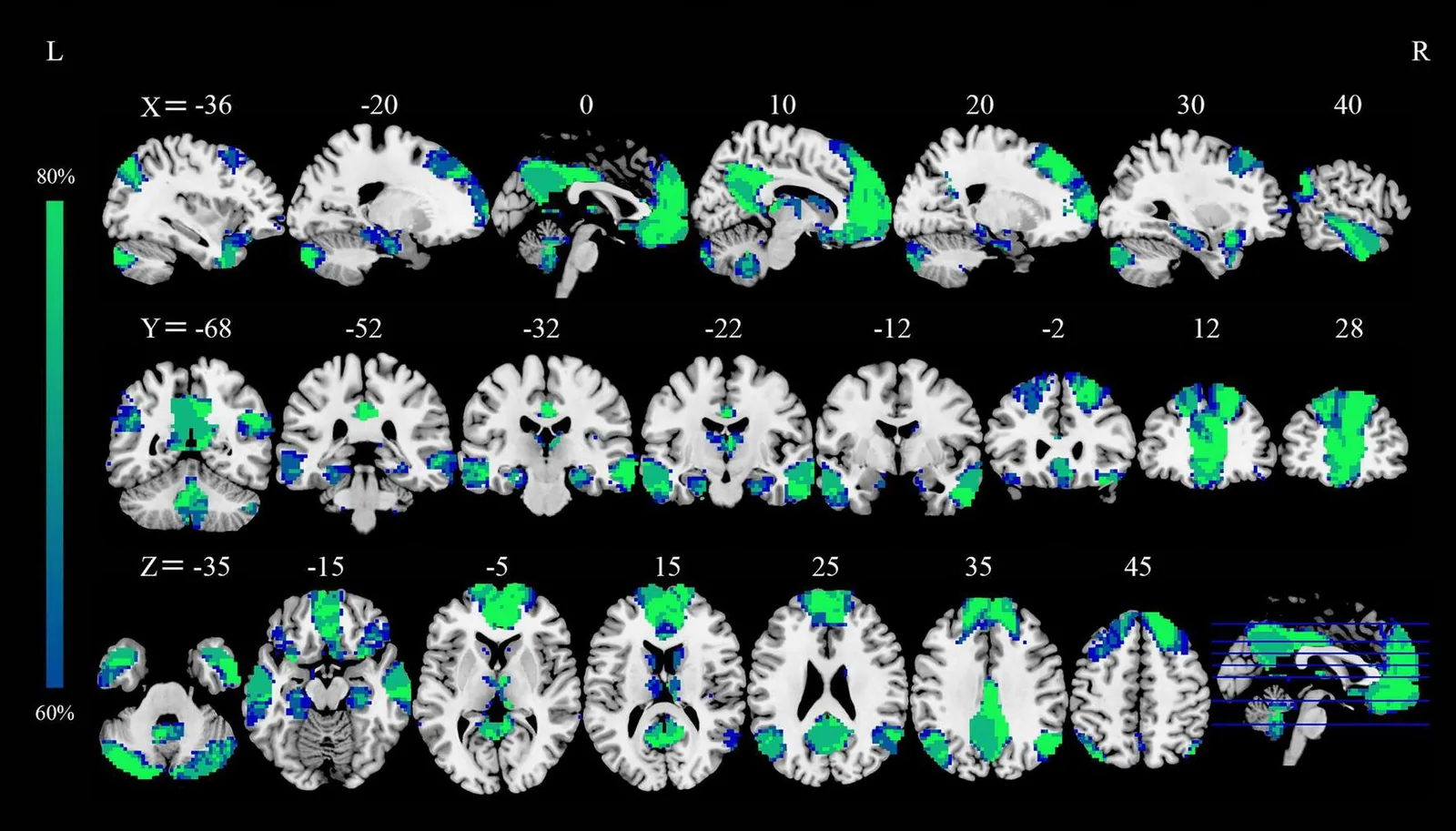
TLE-associated FC overlap maps based on 4-mm radius sphere (HCP). Dysfunctional brain networks are shown as FC probability maps thresholded at 60%, showing brain regions functionally connected to more than 60% of the contrast seeds. TLE, temporal lobe epilepsy; FC, functional connectivity; HCP, Human Connectome Project; L, left; R, right.

By integrating coordinates of reported regional intrinsic neural activity alterations in TLE from the 20 studies with the normative HCP connectome, the FCNM approach revealed convergent functional connectivity associated with TLE. These functional connectivity patterns formed a distributed network with key nodes in the bilateral medial prefrontal cortex (PFC), bilateral precuneus/posterior cingulate cortex (PCC), bilateral angular gyrus, bilateral temporal pole, and bilateral hippocampus (*p* < 0.05, FDR corrected) (Figure 1 and Supplementary Table 4). Cerebellar atlas-based labeling showed that the cerebellar component was located mainly in the bilateral posterior cerebellum, involving Crus I/II and lobule IX regions (Figure 1 and Supplementary Table 4).

### Association with canonical brain networks

**FIGURE 2:**
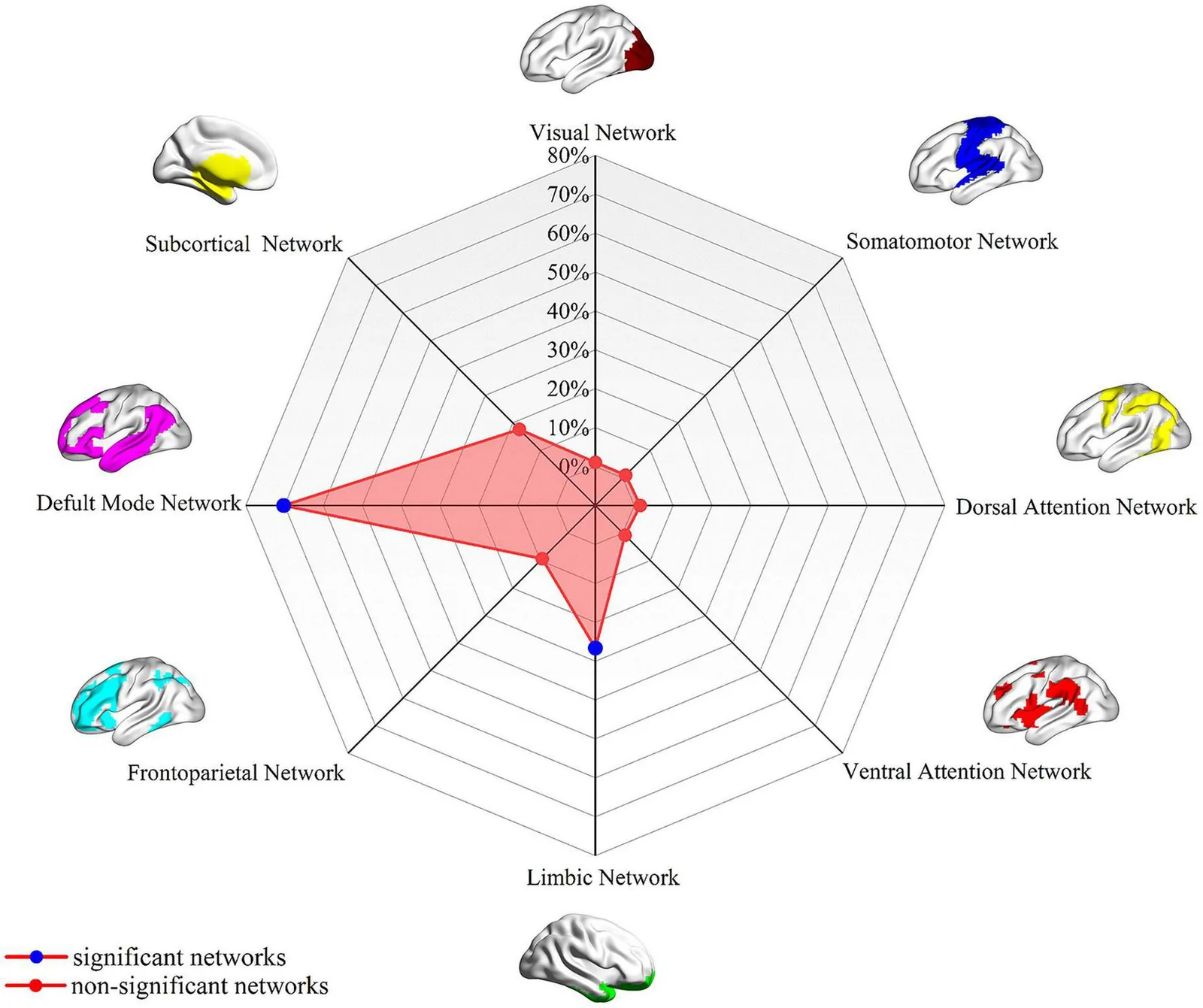
TLE-associated FC overlap maps based on 4-mm radius sphere in association with canonical brain networks (HCP). Polar plots illustrate the proportion of overlapping voxels between each TLE map and a canonical network to all voxels within the corresponding canonical network. The blue dot represents brain dysfunction networks, defined as significant networks, exhibiting ≥20% overlap with canonical networks, whereas the red dot represents non-significant networks with <20% overlap. TLE, temporal lobe epilepsy; FC, functional connectivity; HCP, Human Connectome Project.

Spatial overlap analysis indicated that the TLE-related network preferentially involved specific canonical systems. The network showed the highest overlap with the DMN, largely corresponding to the bilateral medial PFC, the bilateral precuneus/PCC, and the bilateral angular gyrus (overlap proportion: 71.5%). Substantial overlap was also observed with the LN, primarily involving the bilateral temporal pole and hippocampus (26.6%) (Figure 2). Overlap proportions with the remaining canonical networks (visual network, SMN, DAN, VAN, FPN, and subcortical network) were all below 20%. Furthermore, we further used the recently developed NCT to evaluate the correspondence between our TLE regional intrinsic neural activity networks and standard canonical networks. The NCT analysis showed that the TLE functional alteration network predominantly involved the DMN (Dice = 0.656; P_*NCT*_ = 0.001) and LN (Dice = 0.196; P_*NCT*_ = 0.083). Together, both proportional overlap and NCT analyses converged on involvement of the DMN and LN. Furthermore, functional network mapping within the cerebellum revealed that these altered cerebellar voxels preferentially overlapped with the cerebellar representations of the DMN, LN, and FPN.

### Robustness analyses

We performed multiple validation analyses to assess the strength of our results. In the independent SALD validation dataset, the TLE-related convergent functional network partially reproduced the pattern observed in the discovery HCP dataset, with the correspondence being stronger for the DMN component than for the LN component. Quantitatively, the DMN showed moderate and statistically significant spatial correspondence between the HCP and SALD-derived maps (Dice = 0.408; P_*NCT*_ = 0.002), whereas the LN showed weaker, non-significant correspondence (Dice = 0.163; P_*NCT*_ = 0.125). These results indicate that the DMN involvement was robustly replicated across datasets, whereas the limbic involvement was less stable and should be interpreted with caution. Differences between the two datasets are likely attributable, at least in part, to their differing sample sizes (1093 vs. 329) (Supplementary Figures 2, 3). Subsequently, repeating FCNM analyses with seed spheres of 1-mm and 7-mm radii resulted in network patterns that closely resembled those generated using the standard 4-mm radius sphere (Supplementary Figures 4, 5). Furthermore, when replicating the FCNM procedure with spheres of 1-mm and 7-mm radii, the pattern of canonical network involvement remained consistent (Supplementary Figures 6, 7).

We also repeated the FCNM analysis separately for foci with increased and decreased intrinsic activity to examine potential direction-specific patterns. Both direction-specific networks converged prominently onto the DMN. The decreased-alteration network showed strong DMN involvement (Dice = 0.723, P_*NCT*_= 0.001), while the increased-alteration network involved both the DMN (Dice = 0.455, P_*NCT*_ = 0.001) and the SMN (Dice = 0.363, P_*NCT*_ = 0.004). Finally, to evaluate the influence of the overlap threshold used to define the convergent network, we recomputed the FCNM results at 50% and 70% overlap thresholds (based on the HCP dataset with a 4-mm seed radius). At the 50% threshold, the network involved the DMN (Dice = 0.754, P_*NCT*_ = 0.001) and LN (Dice = 0.245, P_*NCT*_ = 0.061), whereas at the more conservative 70% threshold it remained centered on the DMN (Dice = 0.587, P_*NCT*_ = 0.001) and LN (Dice = 0.124, P_*NCT*_ = 0.133).

### Molecular profiles related to the TLE mapping networks

**FIGURE 3:**
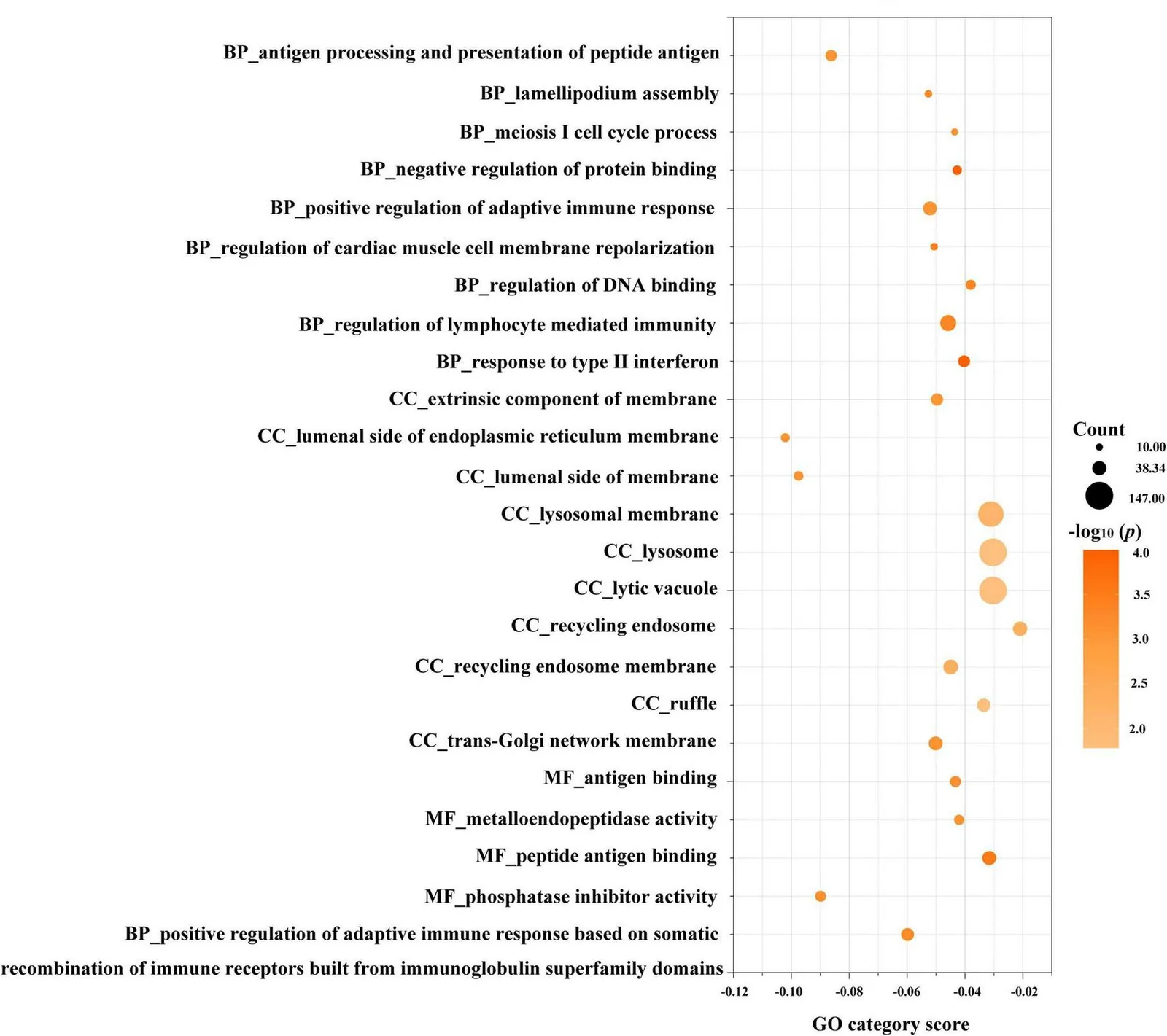
Gene categories associated with the neural correlates of TLE functional network map. TLE, Temporal lobe epilepsy; GO, gene ontology; BP, biological process; CC, cellular component; MF, molecular function.

GO enrichment analysis showed that genes spatially associated with the TLE-related network were enriched for immune-related biological processes (Figure 3 and Supplementary Table 5). Specifically, the analysis highlighted enrichment in Biological Processes (BP) such as antigen processing and presentation of peptide antigen, positive regulation of adaptive immune response, and regulation of lymphocyte mediated immunity. These processes were found to be associated with Cellular Components (CC) integral to protein and antigen trafficking, including the lysosome, recycling endosome, and trans-Golgi network membrane. The underlying drivers for these activities were linked to Molecular Functions (MF) including antigen binding and peptide antigen binding, which are fundamental to immune recognition and response. Furthermore, the enrichment of terms like phosphatase inhibitor activity suggests that these immune pathways are under precise regulatory control.

### Neurotransmitter architecture of tLE-linked network

**FIGURE 4:**
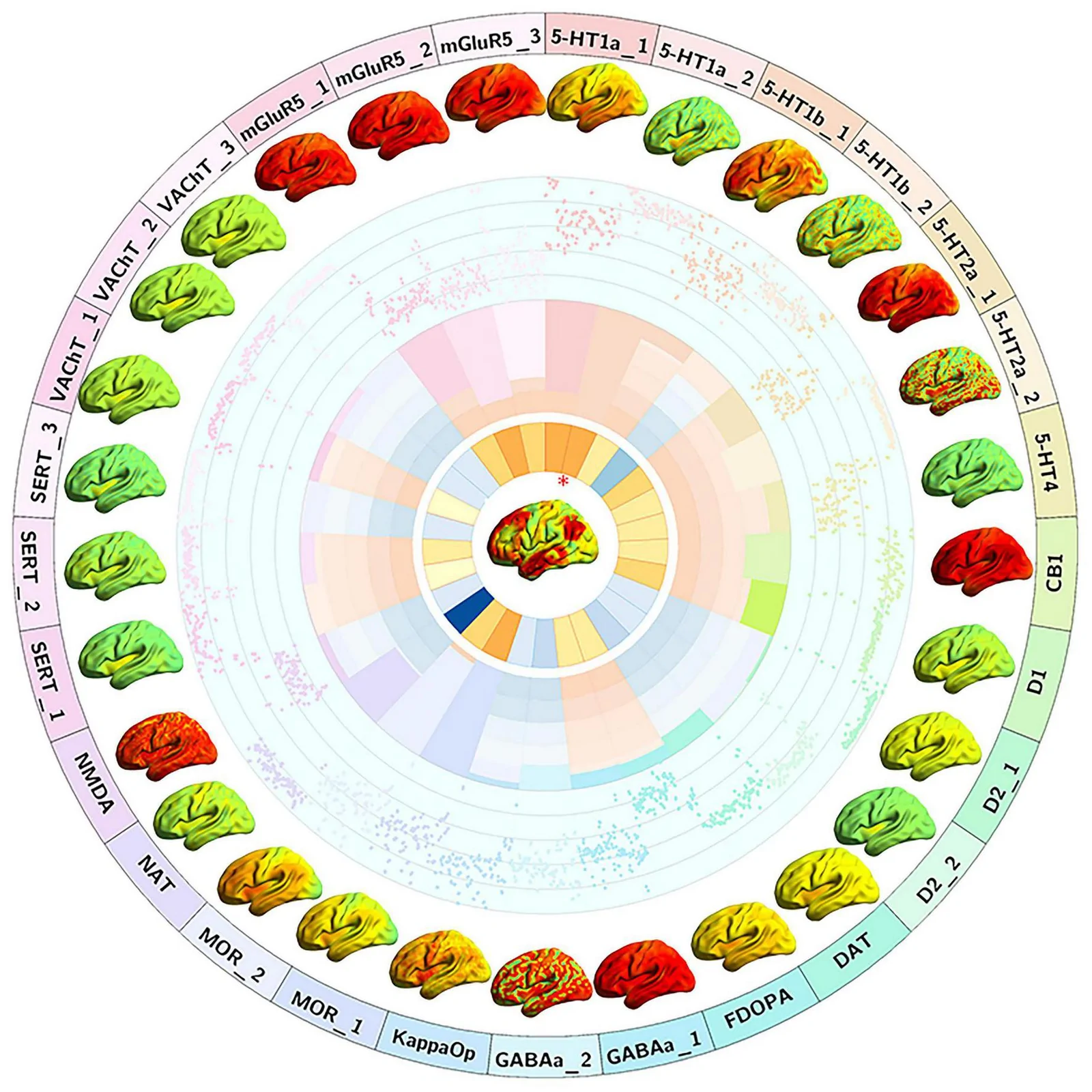
Spatial correlations between the identified TLE-related networks and neurotransmitter distribution maps. *P < 0.05 after false discovery rate (FDR) correction. TLE, temporal lobe epilepsy; 5HT1a, 5-hydroxytryptamine receptor 1A; 5HT1b, 5-hydroxytryptamine receptor 1B; 5HT2a, 5-hydroxytryptamine receptor 2A; 5HT4, 5-hydroxytryptamine receptor 4; CB1, cannabinoid receptor type 1; D1, dopamine receptor D1; D2, dopamine receptor D2; DAT, dopamine transporter; FDOPA, fluorodopa; GABAa, gamma-Aminobutyric acid type A receptor; KappaOp, kappa opioid receptor; MU, mu opioid receptor; NAT, noradrenaline transporter; NMDA, N-methyl-D-aspartate receptor; SERT, serotonin transporter; VAChT, vesicular acetylcholine transporter; mGluR5, metabotropic glutamate receptor 5.

Using the Juspace toolbox (Dukart et al., 2021), the analysis characterized the neurochemical profile of the TLE-associated functional networks by examining its spatial correlations with neurotransmitter system maps (Figure 4 and Supplementary Table 6). These networks exhibited significant positive correlations with 5-hydroxytryptamine receptor 1A (5HT1A; *p* = 0.0221, *r* = 0.2946). Notably, no significant negative correlations were observed with any of the tested neurotransmitter maps (all *p* > 0.05).

## Discussion

By integrating coordinate-based FCNM with large-scale normative connectome data, our study provides novel evidence that heterogeneous findings of regional intrinsic neural activity in TLE converge onto a common brain network. Specifically, these heterogeneous regional changes consistently mapped onto functional networks such as the DMN (the bilateral medial PFC, bilateral precuneus/PCC, and bilateral angular gyrus) and the LN (the bilateral TP and bilateral hippocampus). Because FCNM relies on normative connectomes, the identified network should be interpreted as the normative connectivity profile of reported TLE-related regional abnormalities, rather than as direct evidence of altered functional connectivity in the original patient samples. Similarly, the transcriptomic and neurochemical analyses were based on external normative atlases. Our transcriptomic analysis further revealed that the spatial topography of this network was associated with genes enriched in the adaptive immune response, particularly antigen processing and presentation. Finally, the JuSpace analysis demonstrated significant spatial coupling between these TLE-implicated networks and the distributions of key neurotransmitter receptors, specifically the 5-HT1A.

A primary finding of this study was that TLE-associated regional functional alterations mapped prominently onto the DMN. The DMN, especially the nodes of the medial PFC, angular gyrus and precuneus/PCC identified in our study, plays a crucial role in cognitive and emotional processing, particularly those processes involved in self-referential thinking, autobiographical memory, and emotional regulation (Menon, 2023; Sambuco, 2024). Accumulating evidence supports conceptualizing TLE as a network disorder in which large-scale functional and structural systems, including the DMN, are affected (Bernhardt et al., 2015; Cataldi et al., 2013; Vaughan et al., 2016). Prior studies have reported reduced functional connectivity between DMN hubs, particularly the precuneus/PCC, and medial temporal lobe structures such as the hippocampus (Cook et al., 2019; Haneef et al., 2012, 2014; James et al., 2013). This dysfunction is underpinned by microstructural damage to the connecting white matter tracts (Liao et al., 2011) and GM pathology, ultimately disrupting the DMN’s overall topological efficiency (Yasuda et al., 2015; Zhou et al., 2019). Additional contributors include lesion lateralization (with left TLE tending toward network degradation and right TLE toward complex reorganization) (Haneef et al., 2012) and the preferential involvement of the dorsal DMN subsystem (Doucet et al., 2014). The precuneus/PCC, a central DMN hub, contributes substantially to higher-order integrative functions including self-awareness, episodic memory recall, and introspection (Cui et al., 2018). Convergent evidence consistently indicates that the pathophysiology of TLE involves functional impairment of this core DMN node. Previous studies have reported reduced network centrality and altered spontaneous activity in DMN hubs among patients with TLE (Garcia-Ramos et al., 2016; Qiao et al., 2023). Functional alterations of the angular gyrus in TLE are multifaceted, involving both compensatory task-related hyperactivation (Sidhu et al., 2013) and conflicting patterns of resting-state connectivity (Haneef et al., 2014; Li et al., 2016; Pittau et al., 2012), which highlight its complex role in both pathophysiology and neural reorganization.

Our study also found that regional intrinsic neural activity alterations associated with TLE mapped onto the LN. The limbic system is central to emotion, motivation, social behavior, and memory, particularly memory tied to emotional experience (Kaushal et al., 2024; Mega et al., 1997). It comprises various cortical and subcortical structures (Mota-Rojas et al., 2025); the subcortical components include the amygdala, hippocampus, hypothalamus, mammillary bodies, ventral striatum, and certain thalamic nuclei such as the anterior, intralaminar, and medial dorsal groups (Catani et al., 2013). Previous studies have suggested that limbic structures may constitute important components of the broader pathological network in TLE, with structural disruption potentially propagating along anatomical pathways, (Bonilha et al., 2006) through microstructural damage in key white matter tracts such as the fornix and cingulum (Liacu et al., 2012). Functionally, this structural damage leads to weakened intra-limbic connectivity, aberrant hyper-connectivity with external regions like the DMN (Haneef et al., 2014). Consequently, conceptualizing TLE as a network disorder may help integrate its diverse clinical manifestations and inform the development of network-based therapeutic strategies (Kremen et al., 2025). Our findings indicate that reported TLE-related intrinsic activity abnormalities are located in regions that are normally connected with the temporal pole and hippocampal components of limbic-related circuitry. One study used advanced diffusion MRI techniques to delineate the extensive and complex white matter fiber tracts connecting the TP with the hippocampus, amygdala, orbitofrontal cortex, and insula (Kasa et al., 2022). The hippocampus is a core brain structure essential for forming new episodic memories, learning factual knowledge, and spatial navigation (Joo et al., 2025). Although hippocampal atrophy is a core pathological hallmark, this damage is not an isolated phenomenon but rather the “epicenter” of a broader breakdown within the medial temporal lobe network (Bernasconi et al., 2003). Indeed, the role of the hippocampus transcends that of a local lesion and is now viewed as a “core hub” in a large-scale pathological network. Its dysfunction and connectivity disruptions can lead to secondary atrophy and functional abnormalities in remote brain regions like the thalamus and frontal lobes, ultimately establishing TLE as a “network disease” initiated by the hippocampus that affects whole-brain connectivity and function (Bonilha et al., 2010).

While the DMN constituted the most robust and reproducible component of the TLE-related convergent network across both the discovery and validation datasets, the limbic contribution, although consistent in spatial direction, did not reach statistical significance in the validation cohort. This discrepancy may reflect the greater inter-individual variability and lower signal reliability of limbic regions in resting-state fMRI, as well as the reduced sample size of the validation dataset. Accordingly, the limbic component should be regarded as a suggestive rather than firmly established feature, warranting confirmation in future studies. Direction-specific analyses further showed that DMN involvement was common to both increased and decreased intrinsic activity alterations, underscoring the DMN as the core of the convergent TLE network. The additional SMN involvement observed only for regions of increased activity suggests that direction-specific processes may contribute distinct network features, which merits dedicated investigation in future work.

The transcriptomic analysis indicated that the TLE-related network was spatially associated with normative expression of genes enriched for immune-related and inflammatory biological processes. This complex process begins with the rapid activation of the innate immune system–centered on the IL-1β/IL-1R1 signaling pathway–triggered by seizures or an initial insult (Ravizza et al., 2008; van Gassen et al., 2008). During the chronic phase of the disease, specifically in drug-resistant TLE pathology intensifies with the infiltration of adaptive immune cells (e.g., T lymphocytes) into the brain parenchyma following blood-brain barrier compromise (Toledo et al., 2021). At the molecular level, sustained immune activation in TLE appears to involve immune-related gene expression changes and regulatory mechanisms involving microRNAs and long non-coding RNA networks (Aronica et al., 2010; Che et al., 2024; Wang et al., 2022). Ultimately, these studies have bridged basic pathology and clinical application by establishing clear associations between central inflammation (e.g., elevated brain temperature and metabolic changes) and peripheral immune phenotypes (Mueller et al., 2024). Together, these findings are consistent with the hypothesis that immune and inflammatory mechanisms may be involved in TLE pathophysiology. However, because the AHBA data were obtained from neurologically healthy postmortem donors, our results should not be interpreted as evidence of disease-specific transcriptional alterations in patients with TLE. We used the AHBA because the imaging-transcriptomics framework requires spatially continuous, whole-brain gene-expression data registered to a common stereotaxic space, which is currently best provided by the AHBA (Arnatkeviciute et al., 2019). In contrast, available TLE-specific bulk RNA-seq or single-nucleus RNA-seq datasets are mainly derived from focal resected epileptogenic tissue and therefore lack the whole-brain spatial coverage needed for network-wide spatial correlation (Buchin et al., 2022). Importantly, enrichment of adaptive immune-related pathways should not be interpreted as evidence of T-cell infiltration or active adaptive immune responses in healthy brain parenchyma, since adaptive immune-cell infiltration is a pathological feature of diseased tissue rather than a prominent physiological component of the healthy brain. Rather, our findings represent a hypothesis-generating normative spatial association between the TLE-related functional network and regions with baseline expression of immune-related genes, which requires validation in future patient-level multimodal studies using disease-specific bulk RNA-seq, single-nucleus RNA-seq, or spatial transcriptomic data from epileptogenic tissue.

Furthermore, the spatial distribution of the identified network also showed a significant positive correlation with the normative atlas-based distribution of 5-HT1A receptors. This result should be interpreted as a spatial correspondence with normative serotonergic receptor architecture rather than as direct evidence of altered 5-HT1A receptor availability in patients with TLE. Previous molecular imaging studies have reported reduced 5-HT1A receptor binding in patients with TLE, particularly in limbic regions (Cai et al., 2023; Giovacchini et al., 2005; Savic et al., 2004; Toczek et al., 2003). Our finding is consistent with this literature in that the FCNM-derived network overlaps spatially with regions characterized by higher normative 5-HT1A distribution. This conclusion is not only supported by the highest level of evidence from an integrative meta-analysis (Cai et al., 2023) but is also highly consistent with the findings of multiple pioneering PET studies (Savic et al., 2004; Toczek et al., 2003). Functionally, this receptor reduction is directly linked to exacerbation of seizure activity, as decreased 5-HT1A receptor density correlates negatively with the frequency of interictal spikes (Giovacchini et al., 2005). Therefore, the 5-HT1A association should be considered a hypothesis-generating normative spatial association. Future patient-level PET-fMRI studies are needed to test whether serotonergic abnormalities occur within this network and whether they relate to seizure burden, medication exposure, cognitive dysfunction, or psychiatric comorbidity.

Taken together, our findings extend previous coordinate-based and network-level studies of TLE in several ways. Traditional meta-analytic approaches identify regions where abnormalities are consistently reported across studies, whereas FCNM asks whether heterogeneous abnormalities are connected to a common network. This distinction is particularly important for TLE, where reported rs-fMRI abnormalities vary across imaging metrics, lateralization, clinical characteristics, and preprocessing strategies. By further integrating transcriptomic and neurochemical atlases, the present study links macroscale network localization with normative microscale biological architecture.

### Limitations

Several limitations should be considered when interpreting the findings of this study. First, our analysis relied on coordinate-based data extracted from published studies. Peak coordinates represent summary statistics and lose spatial information compared with full statistical maps or individual participant data (Cheng et al., 2025; Darby et al., 2019). Second, we mapped the networks connected to regional intrinsic neural activity alterations in TLE using a normative connectome from healthy adults. Although this is standard practice in network mapping (Boes et al., 2015; Xu et al., 2024), and evidence suggests that sample selection has minimal impact on network topography, a connectome more closely matched to TLE populations might yield slightly different results. Third, the AHBA gene-expression data were derived from six neurologically healthy postmortem donors. Although AHBA provides the spatially resolved, brain-wide transcriptomic coverage needed for imaging-transcriptomic association analysis, it does not capture disease-specific transcriptional changes in TLE. Available patient-derived TLE RNA-seq and single-nucleus RNA-seq datasets are invaluable for identifying epileptogenic tissue-specific molecular alterations, but they are usually restricted to resected hippocampal or temporal cortical tissue and therefore cannot yet provide whole-brain spatial validation of a distributed FCNM-related network. Consequently, the immune-related enrichment observed here should be interpreted as a normative spatial association and not as direct evidence of adaptive immune infiltration or transcriptional activation in patients with TLE. Future studies combining patient-specific imaging, spatial transcriptomics, and disease-relevant molecular datasets are needed to validate the biological relevance and disease specificity of these findings. Fourth, the input data were heterogeneous with respect to analytic methods and acquisition scanners. While FCNM aims to identify convergence, such heterogeneity may introduce noise and obscure subtle network effects. Fifth, methodological choices such as seed radius and the 60% overlap threshold may influence network definition. Although sensitivity analyses using different seed sizes and overlap threshold supported the robustness of our findings, future studies could explore additional thresholds and analytic strategies (Peng et al., 2022). Sixth, in our initial analysis, we did not separately consider the direction of intrinsic activity alterations, and instead combined increased and decreased coordinates into a single FCNM analysis to identify a direction-independent convergent network; subsequent direction-specific analyses showed that both increased and decreased alterations converged on the DMN, while increased alterations additionally involved the SMN, suggesting partially direction-specific network features. Future studies with larger, direction-stratified datasets are needed to more fully characterize the distinct network significance of increased versus decreased intrinsic activity in TLE. Seventh, our neurotransmitter analyses were correlational and based on normative receptor and transporter atlases derived from healthy individuals. These maps represent typical spatial distributions and do not reflect receptor or transporter alterations in patients with TLE. Thus, our findings indicate spatial association rather than disease-specific neurochemical change. Eighth, although we assessed the robustness of our findings across multiple overlap thresholds and seed sizes, we did not implement spatial null models (e.g., spatially-constrained random coordinates, network-size-matched controls, or coordinates from non-TLE disorders); future studies incorporating such approaches would help further establish the specificity of the observed DMN and limbic convergence. Ninth, the present analysis focused on positive FC and did not include negative correlations. This decision was made because negative correlations are strongly influenced by preprocessing choices, particularly global signal regression, and their biological interpretation remains debated. However, excluding negative correlations may overlook potentially meaningful anticorrelated networks. Future studies should test the robustness of FCNM findings across preprocessing strategies, including with and without GSR, and should examine whether negative connectivity patterns provide additional information about TLE-related network dysfunction. Finally, the present findings may be influenced by publication bias, the absence of spatial null, and the use of normative molecular datasets rather than patient-level disease-specific molecular measurements. Future studies should use image-based or individual-level multimodal data, spatially constrained null models, disorder-control comparisons, and patient-specific molecular or PET measures are needed to validate the disease specificity and biological relevance of these findings.

## Conclusion

In conclusion, this study demonstrates that regional intrinsic neural activity alterations reported across TLE resting-state fMRI studies converge onto a common normative functional network, primarily involving DMN and LN. This network shows spatial associations with normative immune-related transcriptomic profiles and serotonergic neurochemical architecture. These findings support a multi-scale network framework for understanding TLE and generate testable hypotheses for future patient-level multimodal imaging and molecular studies.

## Data Availability

The original contributions presented in this study are included in this article/Supplementary material, further inquiries can be directed to the corresponding authors.

## Footnotes

1
https://www.sdmproject.com/utilities/?show=Coordinates

2
http://human.brain-map.org

3
https://github.com/juryxy/JuSpace
