# Supplementary material for: Convergent functional networks of intrinsic activity alterations in temporal lobe epilepsy and their molecular correlates
Source: Front Mol Neurosci. 2026 Aug 10;19:1909269. doi: 10.3389/fnmol.2026.1909269 (PMC13500546; doi:10.3389/fnmol.2026.1909269)
Supplement: Supplementary file 1 [file Supplementary_file_1.docx]

**Supplementary Materials**

**Convergent functional networks of intrinsic activity alterations in temporal lobe epilepsy and their molecular correlates**

**Search strategy**

Following the PRISMA guidelines, we conducted a comprehensive search of PubMed, Embase, and Web of Science to identify studies reporting alterations in regional intrinsic neural activity, including ALFF, fALFF, dynamic fALFF, ReHo, and dynamic ReHo, in patients with TLE, published between January 1, 2000 and March 5, 2026. The search included keywords as follows:

**Pubmed:** (("Drug Resistant Epilepsy"[MeSH Terms]) OR ("Epilepsy, Temporal Lobe"[MeSH Terms]) OR ("Drug-Resistant Epilepsy"[tiab]) OR ("temporal lobe epilepsy"[tiab]) OR ("TLE"[tiab]) OR ("refractory epilepsy"[tiab]) OR ("intractable epilepsy"[tiab]) OR ("DRE"[tiab])) and ("neuroimaging"[tiab] OR "functional MRI"[tiab] OR "fMRI"[tiab] OR "resting-state"[tiab] OR "ReHo"[tiab] OR "regional homogeneity"[tiab] OR "ALFF"[tiab] OR "amplitude of low-frequency fluctuation"[tiab] OR "fALFF"[tiab] OR "fractional amplitude of low-frequency fluctuations"[tiab])

**Web of science:** TS= (("Temporal Lobe Epilepsy" OR TLE OR "Drug Resistant Epilepsy" OR "refractory epilepsy" OR "intractable epilepsy" OR "DRE")) AND TS= (("Amplitude of Low Frequency Fluctuation*" OR "ALFF" OR "Fractional Amplitude of Low Frequency Fluctuation*" OR "fALFF" OR "Regional Homogeneity" OR "ReHo" OR "Low Frequency Fluctuation*" OR "neuroimaging" OR fMRI OR "functional MRI" OR "resting-state"))

**Embase:** (('temporal lobe epilepsy':ti,ab,kw OR 'refractory epilepsy':ti,ab,kw OR 'resistant epilepsy':ti,ab,kw OR 'intractable epilepsy':ti,ab,kw)) and (("neuroimaging":ti,ab,kw OR "functional MRI":ti,ab,kw OR "fMRI":ti,ab,kw OR "resting-state":ti,ab,kw OR "ReHo":ti,ab,kw OR "regional homogeneity":ti,ab,kw OR "ALFF":ti,ab,kw OR "amplitude of low-frequency fluctuation":ti,ab,kw OR "fALFF":ti,ab,kw OR "fractional amplitude of low-frequency fluctuations":ti,ab,kw))

**Study selection**

All studies were included according to the following criteria: (a) studies that compared ALFF, fALFF or ReHo differences between TLE and healthy controls (HCs) at the whole brain level; (b) reporting coordinates were in Talairach or Montreal Neurological Institute (MNI) space; (c) published as an original paper in English language with peer review; (d) when the three-dimensional standard coordinate details of original manuscripts were not reported, it could be available by making a reasonable request to the corresponding author via email. For longitudinal studies, only baseline data were included.

Exclusion criteria were as follows: (a) were non-original studies (e.g., review, meta-analysis, meeting abstract); (b) patients with TLE had comorbid mental or neurological diagnoses; (c) studies that fail to report whole-brain coordinates; (d) studies based on region-of-interest analyses or using small-volume correction; (e) studies with a sample size less than 10 in each group; (e) the data were covered in another included study in which case the study with the largest sample size or most recent publication was preferentially was selected.

**Steps for transcription processing**

Specifically, the initial procedural step was to refresh the probe-to-gene annotations. The most current information obtained from the National Center for Biotechnology Information was integrated through the use of the Re-Annotator package (1). Furthermore, since expression levels for individual genes were frequently measured by multiple probes, RNA sequencing (RNA-seq) data was used as a reference standard to help choose the most suitable probe for each gene. After filtering out genes not common to both RNA-seq and microarray datasets, we computed the correlation between the two expression measures for the remaining genes. Probes were filtered based on correlation strength, excluding those with r < 0.2 compared to RNA-seq data. When multiple probes corresponded to a single gene, the probe exhibiting the strongest correlation with RNA-seq data was designated as the gene’s representative. A noteworthy constraint in data availability was observed: while data for the left hemisphere was provided by all six donors, only two of them had information available for the right hemisphere Given this pronounced sampling asymmetry, and to avoid the additional bias introduced by the marked differences in gene expression between cortical and subcortical regions (2), we restricted all subsequent analyses to samples from the left cerebral cortex and excluded subcortical samples. The scaled robust sigmoid normalization method was used for within sample (cross-gene) and within-gene (cross-sample) adjustments, with the incorporation of the principle of differential stability (DS). This metric quantifies the consistency of regional gene expression patterns across the donor cohort. Genes were ranked according to their DS, and the top 50% were retained for the primary analysis, for two reasons: first, prior research has reported that genes with higher DS demonstrate more conserved spatial expression patterns and are enriched for brain-related biological functions (3, 4); and second, gene expression conservation across subjects is a prerequisite for transcriptome–neuroimaging spatial correlation analysis. After these processing procedures, we obtained normalized expression data of 5,013 genes for 1,280 tissue samples. Because our functional connectivity network mapping analysis was performed within a gray matter mask, we further restricted our analyses to the samples within this mask, resulting in a final sample × gene matrix of 638 × 5,013.

To explore the genetic underpinnings linked to network-related alterations in TLE, we employed transcriptome-neuroimaging spatial correlation analysis in conjunction with the newly introduced ensemble-based gene category enrichment analysis (GCEA). We initially established a spherical region of interest with a 3 mm radius at the MNI coordinates of each brain tissue sample. Within each sphere, the average t-value was extracted from the statistical map representing correlations between regional intrinsic neural activity alterations and TLE. Subsequently, pearson correlations were calculated for each gene, comparing its expression profile across tissue samples with the corresponding average t-values. This procedure generated 5013 spatial correlation coefficients, which are hereafter designated as “gene scores”. Following a methodology consistent with that of Fulcher et al. (5), the gene scores were then subjected to neuroimaging-spatial ensemble-based GCEA. This analysis began by obtaining the latest GO term hierarchy and annotation data from the GO portal (http://geneontology.org/). The subsequent step required the direct annotation of the 5013 AHBA genes to particular GO categories. We then limited our subsequent analytical work to consider only those GO categories that contained a gene annotation count ranging from 10 to 200. Subsequently, gene scores were aggregated for each GO category; this involved computing the average score of all genes assigned to a particular category. Subsequently, we created 10,000 surrogate maps using the brainsmash package (https://github.com/murraylab/brainsmash). These maps used a spatial-lag model in order to match the spatial autocorrelation structure of the original t-map. Null distributions were created to show the anticipated mean gene scores for each GO category with spatial constraints. This required calculating spatial correlations between the gene expression data and the 10,000 surrogate maps that were created to maintain spatial autocorrelation. Finally, the statistical significance of each GO category was assessed by comparing its empirically derived score against the corresponding ensemble-based null distribution using a two-sided test. To control for multiple comparisons across all tested GO categories, the resulting P-values were corrected using the false discovery rate (FDR, Benjamini–Hochberg procedure), and only categories surviving P_FDR < 0.05 were considered statistically significant. A range of enrichment analyses were carried out for the identified genes associated with network-related alterations in TLE. These genes were functionally annotated using the ToppGene platform (<https://toppgene.cchmc.org/>) (6). Gene Ontology was used to determine their biological functions including molecular function (MF), biological processes (BP), and cellular components (CC).

**Figure S1.** **Flow chart showing the process of study identification and exclusion.**

ROI, regions of interest; TLE, Temporal lobe epilepsy.

**Figure S2. TLE-associated FC overlap maps based on 4-mm radius sphere. (SALD)**

Dysfunctional brain networks are shown as FC probability maps thresholded at 60%, showing brain regions functionally connected to more than 60% of the contrast seeds.

TLE, Temporal lobe epilepsy; FC, functional connectivity; SALD, Southwest University Adult Lifespan Dataset; L, left; R, right.

**Figure S3. TLE-associated** **FC overlap maps based on 4-mm radius sphere in association with canonical brain networks. (SALD)**

Polar plots illustrate the proportion of overlapping voxels between each TLE map and a canonical network to all voxels within the corresponding canonical network. Note: The blue dot represents brain dysfunction networks, defined as significant networks, exhibiting ≥20% overlap with canonical networks, whereas the red dot represents non-significant networks with <20% overlap.

TLE, Temporal lobe epilepsy; FC, functional connectivity; SALD, Southwest University Adult Lifespan Dataset.

**Figure S4. TLE-associated FC overlap maps based on 1-mm radius sphere. (HCP)**

Dysfunctional brain networks are shown as FC probability maps thresholded at 60%, showing brain regions functionally connected to more than 60% of the contrast seeds.

TLE, Temporal lobe epilepsy; FC, functional connectivity; HCP, Human Connectome Project; L, left; R, right.

**Figure S5. TLE-associated FC overlap maps based on 7-mm radius sphere. (HCP)**

Dysfunctional brain networks are shown as FC probability maps thresholded at 60%, showing brain regions functionally connected to more than 60% of the contrast seeds.

TLE, Temporal lobe epilepsy; FC, functional connectivity; HCP, Human Connectome Project; L, left; R, right.

**Figure S6. TLE-associated** **FC overlap maps based on 1-mm radius sphere in association with canonical brain networks. (HCP)**

Polar plots illustrate the proportion of overlapping voxels between each TLE map and a canonical network to all voxels within the corresponding canonical network. Note: The blue dot represents brain dysfunction networks, defined as significant networks, exhibiting ≥20% overlap with canonical networks, whereas the red dot represents non-significant networks with <20% overlap.

TLE, Temporal lobe epilepsy; FC, functional connectivity; HCP, Human Connectome Project.

**Figure S7. TLE-associated** **FC overlap maps based on 7-mm radius sphere in association with canonical brain networks. (HCP)**

Polar plots illustrate the proportion of overlapping voxels between each TLE map and a canonical network to all voxels within the corresponding canonical network. Note: The blue dot represents brain dysfunction networks, defined as significant networks, exhibiting ≥20% overlap with canonical networks, whereas the red dot represents non-significant networks with <20% overlap.

TLE, Temporal lobe epilepsy; FC, functional connectivity; HCP, Human Connectome Project.

**Table S1. Demographic information of the discovery and validation datasets.**

| **Dataset** | **Sample size** | **Age (years)** | **Gender (F/M)** |
| --- | --- | --- | --- |
| HCP | 1093 | 28.78 ± 3.69 | 594/499 |
| SALD | 329 | 37.81 ±13.79 | 207/122 |

Age is expressed as mean ± standard deviation (mean ± SD).

F, female; HCP, Human Connectome Project; M, male; SALD, Southwest University Adult Lifespan Dataset.

**Table S2. Resting-state fMRI parameters of discovery and validation datasets.**

| **Parameter** | **HCP** | **SALD** |
| --- | --- | --- |
| Scanner | 3.0T Siemens Trio | 3.0T Siemens Trio |
| Sequence | GRE-EPI | GRE-EPI |
| TR (ms) | 720 | 2000 |
| TE (ms) | 33.1 | 30 |
| FA (°) | 52 | 90 |
| FOV (mm²) | 208×180 | 220×220 |
| Matrix size | 104×90 | 64×64 |
| Slice thickness (mm) | 2 | 3 |
| Slice gap (mm) | 0 | 1 |
| Slices | 72 | 32 |
| Time points | 1210 | 242 |

FA, flip angle; fMRI, functional magnetic resonance imaging; FOV, field of view; GRE-EPI, gradient-recalled echo-Planar Imaging; HCP, Human Connectome Project; SALD, Southwest University Adult Lifespan Dataset; TE, echo time; TR, repetition time.

**Table S3. Demographic information of the six adult donors in the AHBA.**

| **Donor ID** | **Sex** | **Age (years)** | **Ethnicity** | **Hemisphere** |
| --- | --- | --- | --- | --- |
| H0351.1009 | Male | 57 | Caucasian | Left |
| H0351.1012 | Male | 31 | Caucasian | Left |
| H0351.1015 | Female | 49 | Hispanic | Left |
| H0351.1016 | Male | 55 | Caucasian | Left |
| H0351.2001 | Male | 24 | African American | Both |
| H0351.2002 | Male | 39 | African American | Both |

AHBA, Allen Human Brain Atlas.

**Table S4. Brain FC with altered regional neural activity in TLE.**

| **Brain regions** | **R/L** | **Voxels** | **Brodmann areas** | **X** | **Y** | **Z** |
| --- | --- | --- | --- | --- | --- | --- |
| medial prefrontal cortex (PFC) | L | 580 | BA8/9/10/11 | -17 | 37 | 48 |
| medial prefrontal cortex (PFC) | R | 559 | BA8/9/10/11 | 17 | 37 | 48 |
| precuneus/posterior cingulate cortex (PCC) | L | 464 | BA23/30 | -13 | -53 | 24 |
| precuneus/posterior cingulate cortex (PCC) | R | 487 | BA23/30 | 8 | -53 | 33 |
| angular gyrus | L | 355 | BA39 | -38 | -75 | 44 |
| angular gyrus | R | 300 | BA39 | 41 | -75 | 45 |
| temporal pole | L | 310 | BA38 | -46 | 6 | -35 |
| temporal pole | R | 293 | BA38 | 50 | 6 | -33 |
| hippocampus | L | 90 | BA20/37/30 | -31 | -27 | -16 |
| hippocampus | R | 110 | BA20/37/30 | 22 | -27 | 9 |
| cerebellar Crus I/II | L | 482 | - | -39 | -74 | -42 |
| cerebellar Crus I/II | R | 646 | - | 41 | -75 | -42 |
| cerebellar lobule IX | L | 97 | - | -12 | -49 | -42 |
| cerebellar lobule IX | R | 134 | - | 8 | -52 | -42 |

BA, Brodmann area; FC, functional connectivity; TLE, Temporal lobe epilepsy; L, left; R, right.

**Table S5. Enrichment analysis of the genes in the identified TLE functional network.**

| **GO name** | **ID label** | **Ontology** | **GO category score** | **Description** | **p-value** | **count** |
| --- | --- | --- | --- | --- | --- | --- |
| antigen processing and presentation of peptide antigen | 0048002 | BP | -0.086237733 | The process by which antigen-presenting cells process peptide antigens and display them on the cell surface bound to MHC molecules for immune recognition. | 0.0008 | 25 |
| lamellipodium assembly | 0030032 | BP | -0.052635126 | Formation of a lamellipodium, a thin sheetlike extension of the surface of a migrating cell. | 0.0005 | 11 |
| meiosis I cell cycle process | 0061982 | BP | -0.043574127 | A process that contributes to the first meiotic division. The first meiotic division is the reductive division resulting in the separation of homologous chromosome pairs. | 0.0008 | 10 |
| negative regulation of protein binding | 0032091 | BP | -0.042722787 | Any process that stops, prevents, or reduces the frequency, rate or extent of protein binding. | 0.0001 | 17 |
| positive regulation of adaptive immune response | 0002821 | BP | -0.052098069 | Any process that activates or increases the frequency, rate, or extent of an adaptive immune response. | 0.0008 | 37 |
| regulation of cardiac muscle cell membrane repolarization | 0099623 | BP | -0.050678927 | Any process that modulates the establishment or extent of a change in membrane potential in the polarizing direction towards the resting potential in a cardiomyocyte. | 0.0004 | 11 |
| regulation of DNA binding | 0051101 | BP | -0.038029958 | Any process that modulates the frequency, rate or extent of DNA binding. DNA binding is any process in which a gene product interacts selectively with DNA (deoxyribonucleic acid). | 0.0005 | 20 |
| regulation of lymphocyte mediated immunity | 0002706 | BP | -0.045833322 | Any process that modulates the frequency, rate, or extent of lymphocyte mediated immunity. | 0.0005 | 49 |
| response to type II interferon | 0034341 | BP | -0.040317628 | Cellular responses triggered by interferon-gamma (IFN-γ), including changes in gene expression and cell activity. | 0.0001 | 27 |
| extrinsic component of membrane | 0019898 | CC | -0.049668936 | The component of a membrane consisting of gene products and protein complexes that are loosely bound to one of its surfaces, but not integrated into the hydrophobic region. | 0.0009 | 29 |
| lumenal side of endoplasmic reticulum membrane | 0098553 | CC | -0.102092821 | The leaflet of the endoplasmic reticulum membrane that faces the lumen, including any protein embedded in, attached to, or peripherally associated with it. | 0.0009 | 16 |
| lumenal side of membrane | 0098576 | CC | -0.097525756 | The leaflet of an organelle membrane that faces the lumen, including any protein embedded in, attached to, or peripherally associated with it. | 0.0008 | 18 |
| lysosomal membrane | 0005765 | CC | -0.0310688 | The lipid bilayer surrounding the lysosome and separating its contents from the cell cytoplasm. | 0.0067 | 124 |
| lysosome | 0005764 | CC | -0.030370885 | A membrane-bound organelle containing hydrolytic enzymes that digest and recycle cellular components. | 0.0167 | 147 |
| lytic vacuole | 0000323 | CC | -0.030370885 | A vacuole that is maintained at an acidic pH and which contains degradative enzymes, including a wide variety of acid hydrolases. | 0.0167 | 147 |
| recycling endosome | 0055037 | CC | -0.020983262 | An organelle consisting of a network of tubules that functions in targeting molecules, such as receptors transporters and lipids, to the plasma membrane. | 0.0044 | 39 |
| recycling endosome membrane | 0055038 | CC | -0.044950133 | The lipid bilayer surrounding a recycling endosome. | 0.0047 | 42 |
| ruffle | 0001726 | CC | -0.033543229 | Projection at the leading edge of a crawling cell; the protrusions are supported by a microfilament meshwork. | 0.0155 | 35 |
| trans-Golgi network membrane | 0032588 | CC | -0.050143144 | The lipid bilayer surrounding any of the compartments that make up the trans-Golgi network. | 0.0008 | 37 |
| antigen binding | 0003823 | MF | -0.043304259 | Selective binding to antigens, enabling immune recognition and response. | 0.0007 | 24 |
| metalloendopeptidase activity | 0004222 | MF | -0.042025293 | Catalyzes the hydrolysis of peptide bonds in proteins using metal ions and water. | 0.0008 | 20 |
| peptide antigen binding | 0042605 | MF | -0.031629805 | Binding to an antigen peptide. | 0.0003 | 38 |
| phosphatase inhibitor activity | 0019212 | MF | -0.08989488 | Binds to and stops, prevents or reduces the activity of a phosphatase. | 0.0007 | 23 |
| positive regulation of adaptive immune response based on somatic recombination of immune receptors built from immunoglobulin superfamily domains | 0002824 | BP | -0.059847365 | Enhances or activates adaptive immune responses mediated by antigen-specific immune receptors. | 0.0006 | 32 |

BP, biological processes; CC, cellular components; GO, gene ontolog**y**; MF, molecular function; TLE, temporal lobe epilepsy.

**Table S6. Neurotransmitter Profiles Underlying Functional Network Alterations in TLE.**

| **PET Map** | **Mean Fisher's z (Pearson r)** | **p** |
| --- | --- | --- |
| 5HT1a_1 | 0.2182 | 0.08338 |
| 5HT1a_2 | 0.2946 | 0.02219 |
| 5HT1b_1 | 0.0426 | 0.76625 |
| 5HT1b_2 | -0.069 | 0.45791 |
| 5HT2a_1 | 0.0899 | 0.32434 |
| 5HT2a_2 | 0.1148 | 0.39212 |
| 5HT4 | 0.0498 | 0.60248 |
| CB1 | 0.1089 | 0.43971 |
| D1 | 0.0871 | 0.34853 |
| D2_1 | -0.008 | 0.93461 |
| D2_2 | -0.005 | 0.95581 |
| DAT | -0.051 | 0.58028 |
| FDOPA | 0.0325 | 0.73525 |
| GABAa_1 | 0.0356 | 0.69166 |
| GABAa_2 | -0.061 | 0.64407 |
| KappaOp | 0.0151 | 0.91262 |
| MOR_1 | 0.2896 | 0.04539 |
| MOR_2 | 0.2563 | 0.13197 |
| NAT | -0.154 | 0.08818 |
| NMDA | -0.020 | 0.88642 |
| SERT_1 | 0.0017 | 0.98740 |
| SERT_2 | 0.0354 | 0.70586 |
| SERT_3 | -0.003 | 0.97241 |
| VAChT_1 | 0.0236 | 0.79824 |
| VAChT_2 | -0.021 | 0.82044 |
| VAChT_3 | -0.002 | 0.97901 |
| mGluR5_1 | 0.1683 | 0.20576 |
| mGluR5_2 | 0.2221 | 0.09958 |
| mGluR5_3 | 0.1843 | 0.17117 |

TLE, temporal lobe epilepsy; 5HT1a, 5-HydroxyTryptamine 1A receptor (Serotonin 1A receptor); 5HT1b, 5-HydroxyTryptamine 1B receptor (Serotonin 1B receptor); 5HT2a, 5-HydroxyTryptamine 2A receptor (Serotonin 2A receptor); 5HT4, 5-HydroxyTryptamine 4 receptor (Serotonin 4 receptor); CB1, Cannabinoid receptor type 1; D1, Dopamine D1 receptor; D2, Dopamine D2 receptor; DAT, Dopamine Transporter; FDOPA, Fluoro-L-DOPA (DOPA decarboxylase substrate / presynaptic dopamine synthesis); GABAa, Gamma-Aminobutyric acid type A receptor; KappaOp, Kappa Opioid receptor; MOR, Mu Opioid Recepto; NAT, Noradrenaline Transporter; NMDA, N-methyl-D-aspartate receptor; SERT, Serotonin Transporter; VAChT, Vesicular AcetylCholine Transporter; mGluR5, Metabotropic Glutamate Receptor 5.

**References**

1.	Arloth J, Bader DM, Röh S, Altmann A. Re-Annotator: Annotation Pipeline for Microarray Probe Sequences. PLoS One. 2015;10(10):e0139516.

2.	Hawrylycz MJ, Lein ES, Guillozet-Bongaarts AL, Shen EH, Ng L, Miller JA, et al. An anatomically comprehensive atlas of the adult human brain transcriptome. Nature. 2012;489(7416):391-9.

3.	Hawrylycz M, Miller JA, Menon V, Feng D, Dolbeare T, Guillozet-Bongaarts AL, et al. Canonical genetic signatures of the adult human brain. Nature neuroscience. 2015;18(12):1832-44.

4.	Fulcher BD, Arnatkeviciute A, Fornito A. Overcoming false-positive gene-category enrichment in the analysis of spatially resolved transcriptomic brain atlas data. Nature communications. 2021;12(1):2669.

5.	Fulcher BD, Arnatkeviciute A, Fornito A. Overcoming false-positive gene-category enrichment in the analysis of spatially resolved transcriptomic brain atlas data. Nat Commun. 2021;12(1):2669.

6.	Chen J, Bardes EE, Aronow BJ, Jegga AG. ToppGene Suite for gene list enrichment analysis and candidate gene prioritization. Nucleic Acids Res. 2009;37(Web Server issue):W305-11.
